# Unique Presentation of the Follicular Occlusion Tetrad: A Case Report and Review of the Literature

**DOI:** 10.7759/cureus.90254

**Published:** 2025-08-16

**Authors:** Robert Adler, Isha Gandhi, Maisha Sheikh, Justin Marson, Steven Fishman

**Affiliations:** 1 Dermatology, State University of New York (SUNY) Downstate College of Medicine, New York City, USA; 2 Medicine, University of Minnesota Twin Cities Medical School, Minneapolis, USA; 3 Dermatology, Mount Sinai Medical Center, New York City, USA

**Keywords:** acne conglobata, case report, dissecting cellulitis of scalp, follicular occlusion tetrad, glp-1 agonist, hidradenitis suppurativa, literature review, pilonidal cyst

## Abstract

The follicular occlusion tetrad is a chronic inflammatory complex that consists of four co-related conditions in which follicles are blocked with keratin rupture and lead to inflammation. These conditions include hidradenitis suppurativa (HS), acne conglobata (AC), dissecting cellulitis of the scalp (DCS), and pilonidal cyst (PC). Our unique case reports a 43-year-old female patient who presented with a 36-year history of all four elements of the tetrad after previous treatment with ciprofloxacin, spironolactone, and salicylic acid. Our case is notable as the patient presented after being on semaglutide for weight loss, as well as the first report of the full tetrad in a female patient. A comprehensive review of the follicular occlusion tetrad was conducted using current literature. The goal of the literature review was to provide a general overview of the patient population diagnosed with follicular occlusion tetrad. Additionally, weight loss medications were discussed as potential treatments for patients given the link between the disease and obesity. Further study is warranted to investigate the efficacy of newer therapies in managing patients.

## Introduction

The follicular occlusion tetrad consists of four co-related conditions in which follicles are blocked with keratin rupture and lead to inflammation [1]. The tetrad, which comprises hidradenitis suppurativa (HS), acne conglobata (AC), dissecting cellulitis of the scalp (DCS), and pilonidal cyst (PC), often occurs as independent conditions, though combined presentations have been reported [2]. Pillsbury and Shelley are often credited with coining the term “follicular occlusion triad” in 1954, with pilonidal cyst being recognized in 1975 [3,4]. Reports of patient presentations meeting all four criteria of the follicular occlusion tetrad are rare. A meta-analysis of follicular occlusion triad and tetrad cases revealed that 88% of cases were present in men, with just over a majority being African American [5]. A combination of environmental factors have been discussed, and genetic factors such as KRT17 mutations have been explored [6,7]. Though encountering one or more of these conditions may be common, their combined presentation warrants further discussion.

In this report, we report a rare case of follicular occlusion tetrad in a middle-aged female patient who presented with all four conditions while undergoing treatment for weight loss.

## Case presentation

**Figure 1:**
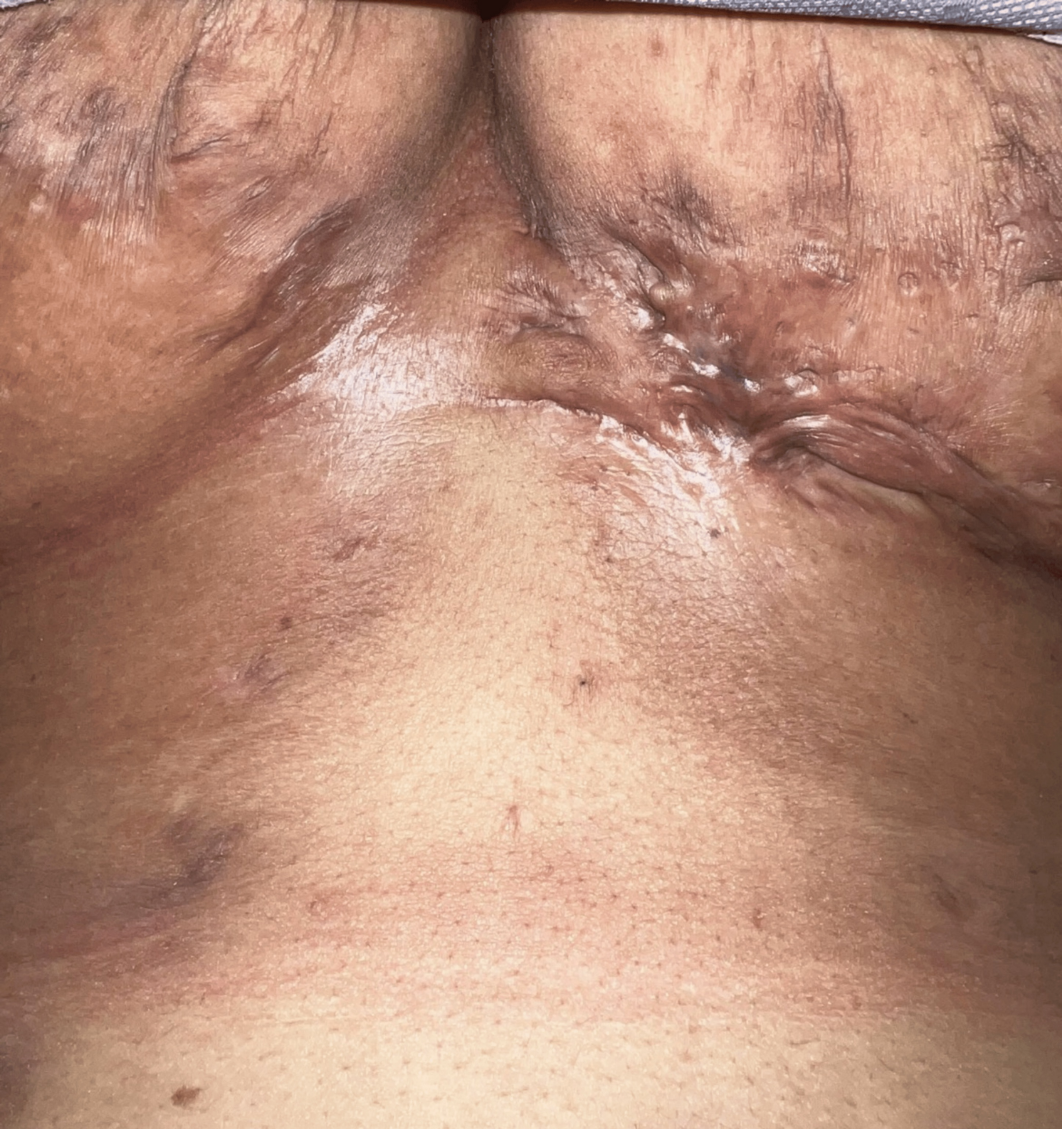
Hidradenitis suppurativa: strand-like scarring below breasts

**Figure 2:**
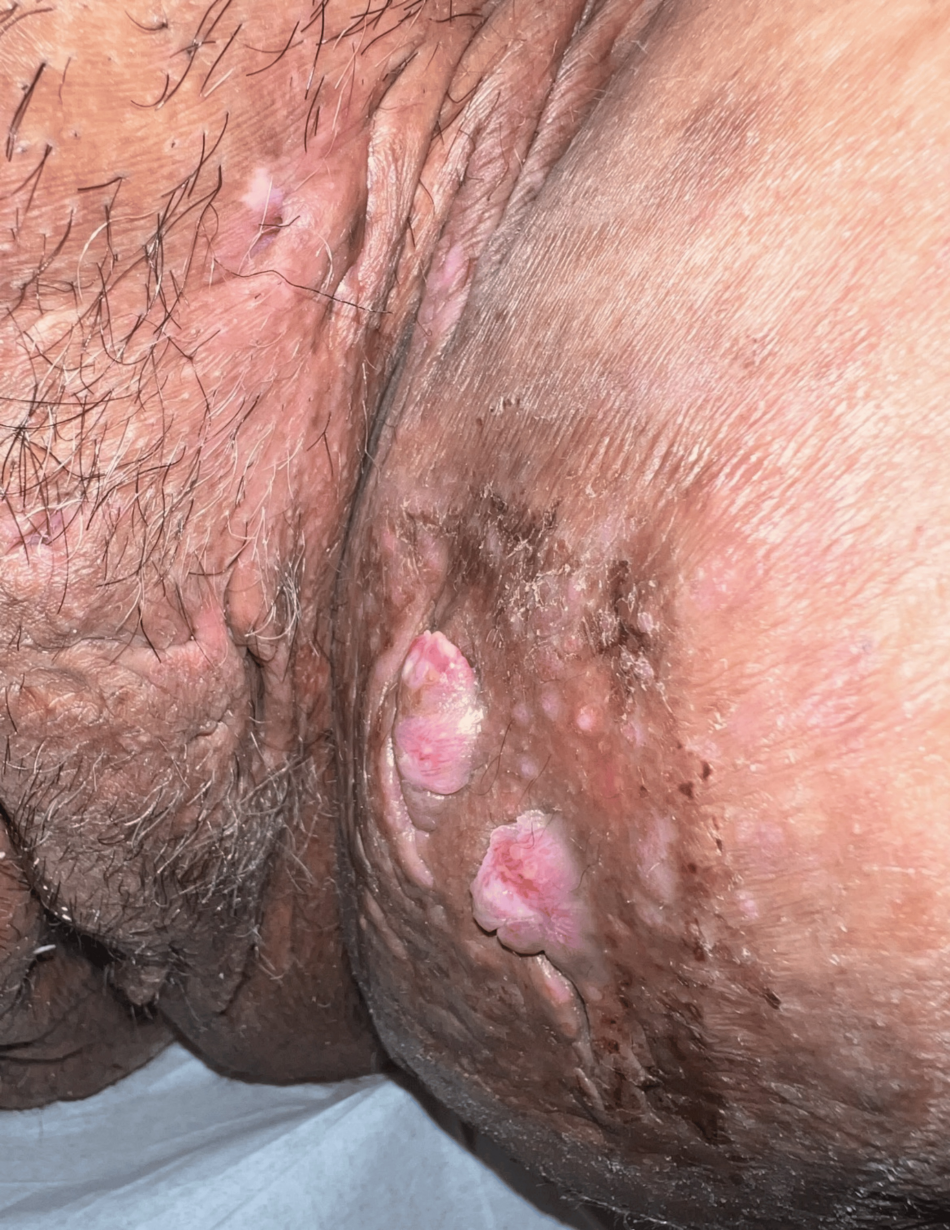
Hidradenitis suppurativa - lesions in the axillary region

**Figure 3:**
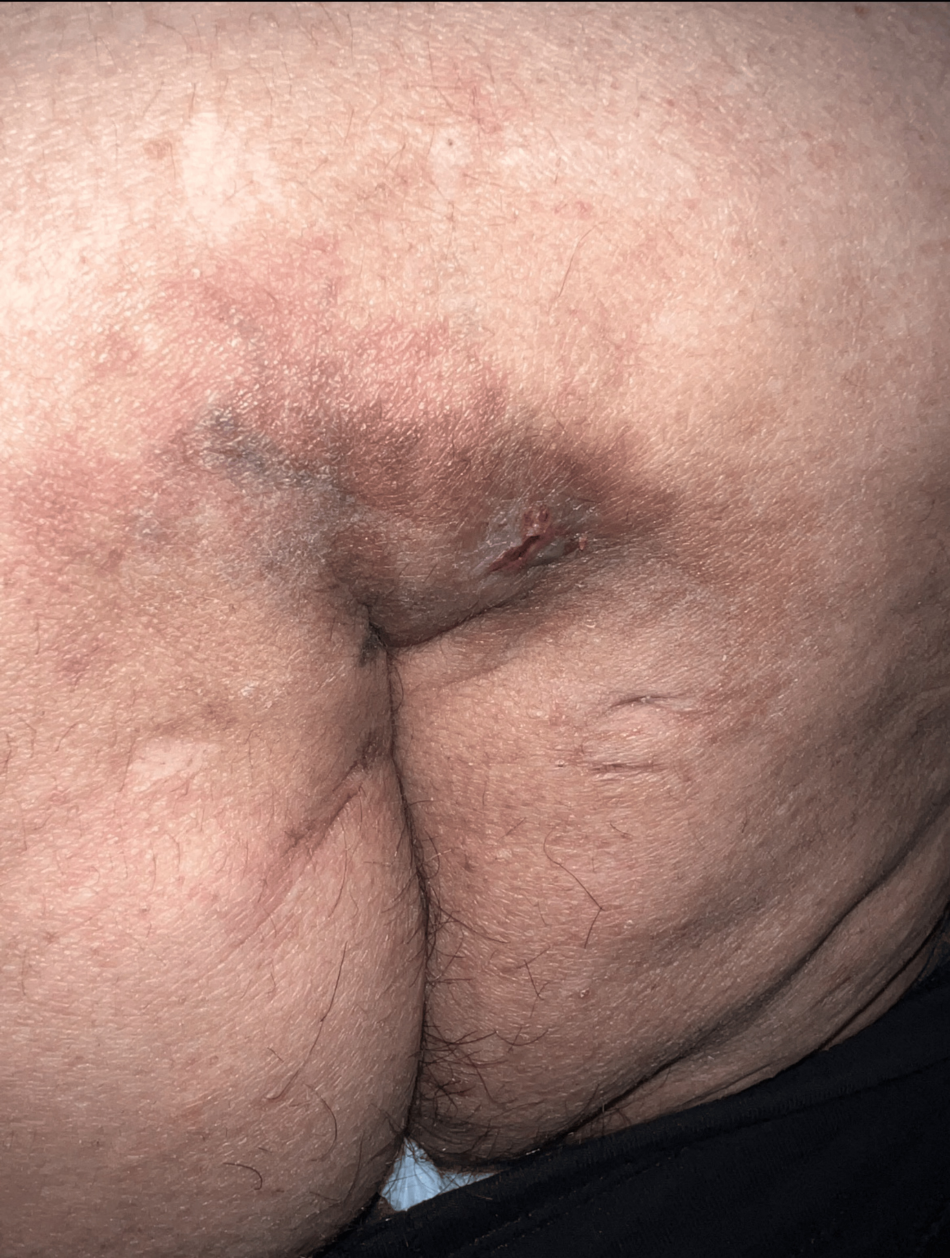
Pilonidal cyst in the intergluteal region

**Figure 4:**
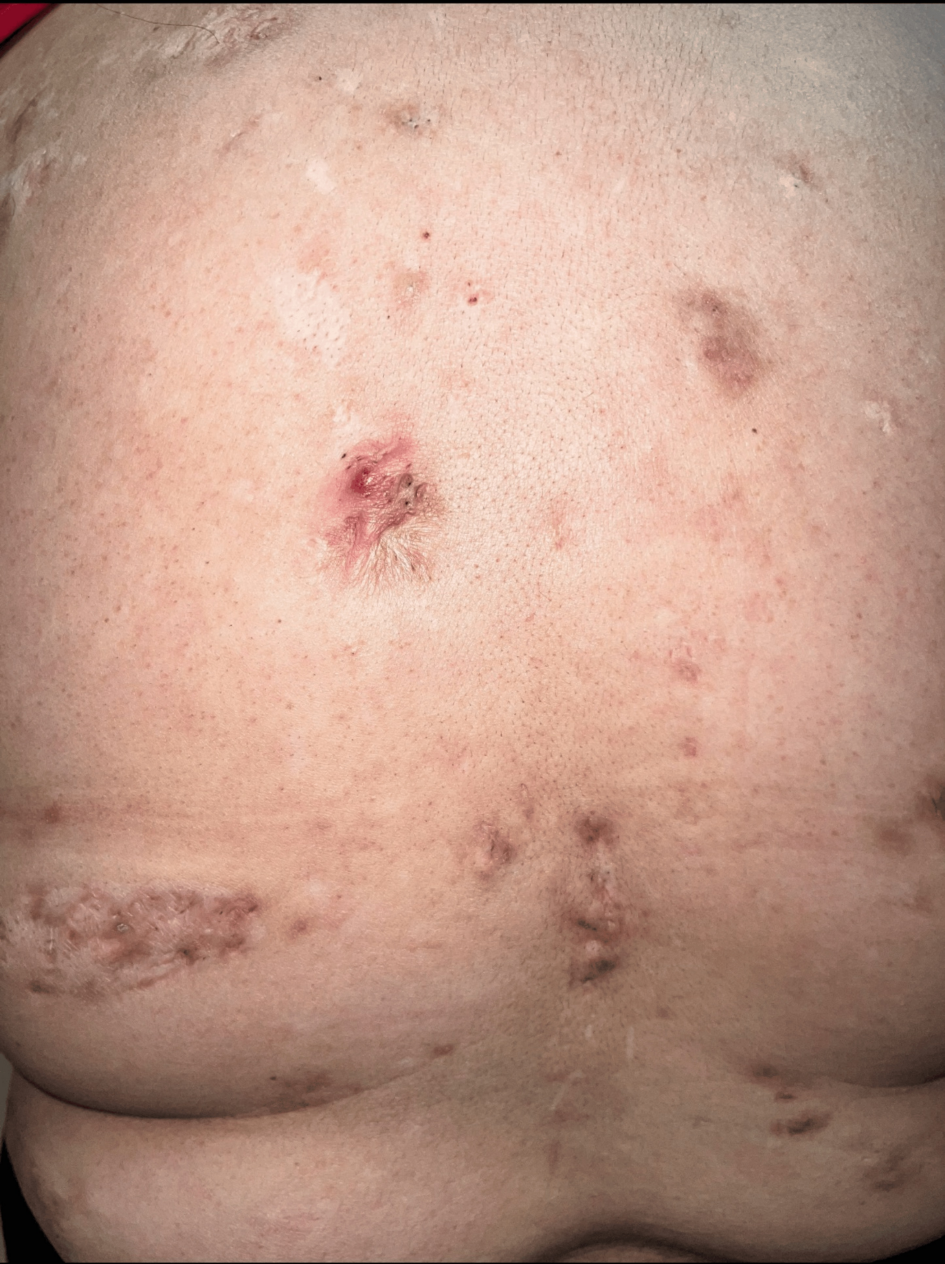
Acne conglobata: tunneling suppurative lesions on the back

**Figure 5:**
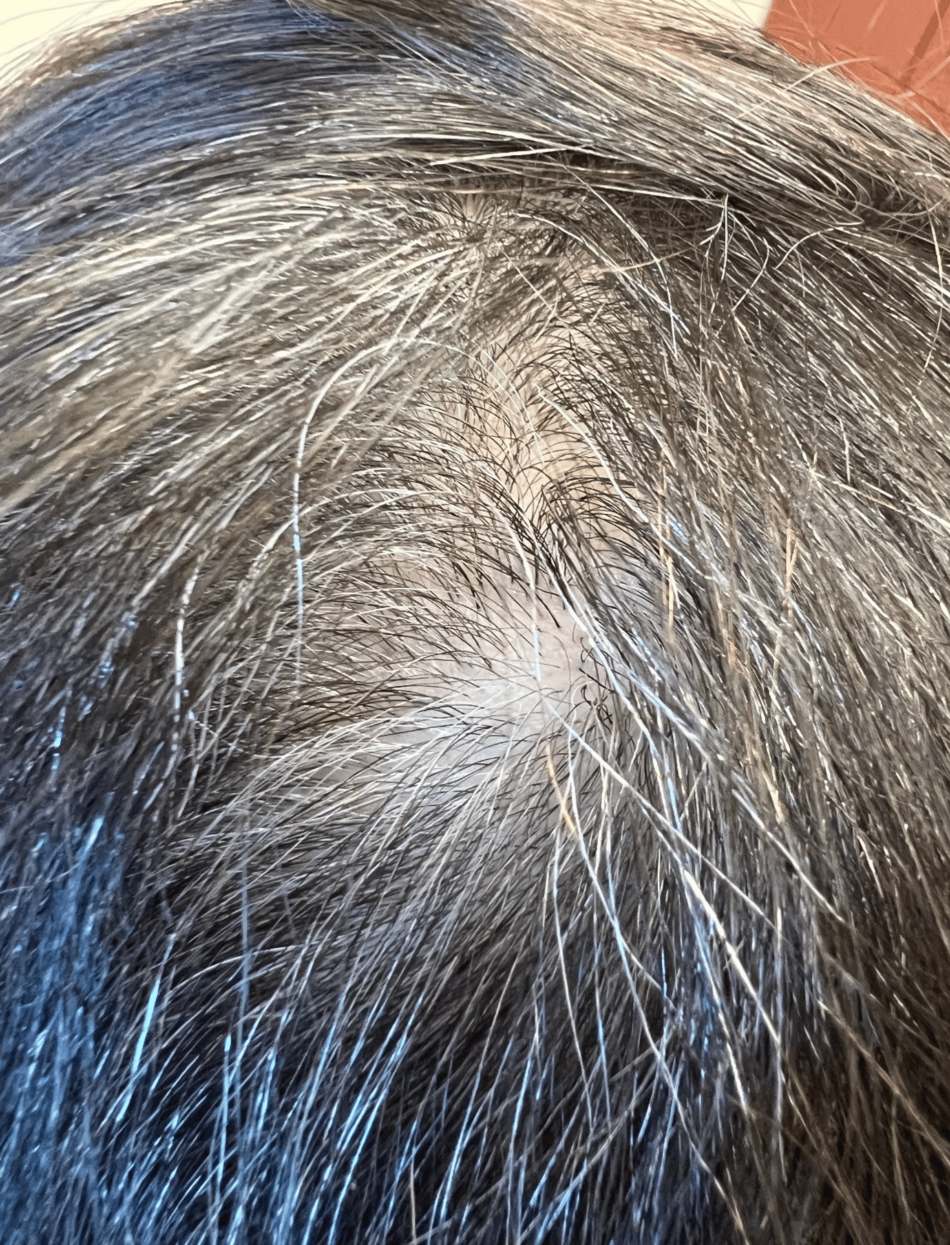
Dissecting cellulitis of the scalp

A 43-year-old woman presented with a chief complaint of generalized painful boils occurring since the age of seven previously managed with ciprofloxacin, spironolactone, and salicylic acid. Her personal medical history is notable for obesity, which has been medically managed with semaglutide as well as hypertension. Multiple verrucous papules, xerosis, and abscessed cysts were noted upon examination. Band-like scars were noted inferior to the left breast (Figures 1, 2), pilonidal cysts were detected in the intergluteal region (Figure 3), and burrowing abscesses on the back were detected (Figure 4). Additionally, mild cellulitis of the scalp was observed (Figure 5). The patient was subsequently diagnosed with all four manifestations of the follicular occlusion tetrad. An attempt was made to begin secukinumab therapy; subsequently, the patient was lost to follow-up.

## Discussion

Methods

A database search was performed of PubMed and Google Scholar with the search term “follicular occlusion tetrad” (n=523). Titles and abstracts were screened for relevance, and duplicates were removed. Articles from the years 1950 to 2024 were included. Additionally, case reports that addressed the tetrad were included, whereas those discussing the triad were excluded. A final cohort of nine published follicular occlusion tetrad case reports was included for review. Metrics analyzed included patient age, sex, risk factors, treatment history, and response to treatment.

Results

The results are shown in Table 1. Of 10 follicular occlusion tetrad cases, patients were on average 33.5 years old (range 19-50 years). Nine out of 10 patients were men. Eight patients were classified as either overweight or obese. Genetic or family history was collected from three patients, of which two had a KRT17 mutation and one patient reported a family history of follicular occlusion disorders. Six patients underwent treatment with relapse before presentation. A variety of antibiotics, steroids, retinoids, and procedural therapies were noted to be used in seven patients, of which two patients relapsed after treatment [2,6,8-14].

**Table 1:** Characteristics of Cases of Follicular Occlusion Tetrad Reported in the Literature

Follicular Occlusion Tetrad Clinical Findings	
Age median (range) years	33.5 (19-50)
Male, n (%)	9 (90%)
Female, n (%)	1 (10%)
Overweight/Obese (n)	8
Genetic History (n)	3
Prior Treatment Before Presentation	6
Treatment Initiated	6
Treatment Outcomes	Successful: 3
	Successful Followed by Relapse: 1
	Unsuccessful: 1

A summary of previous cases of follicular occlusion tetrad, including risk factors, treatments, and clinical progress, is presented in Table 2. 

**Table 2:** Published cases (in English language) of Follicular Occlusion Tetrad since 1950 FOT: Follicular occlusion tetrad; T2DM: type 2 diabetes mellitus.

Study (Author, Year)	Patient Age and Sex		Risk Factors	Previous Treatment	Treatment Initiated	Progress
Ng-Wong et al., 2023 [2]	22-year-old male		Unknown	Incision and Drainage	Antibiotics (Clindamycin, Rifampin, Trimethoprim-sulfamethoxazole, Minocycline, and Amoxicillin-clavulanate), Dapsone, Isotretinoin, Excisional debridement, Flap Procedure, Skin Grafting	Incisions in the face, abdomen, groin, and scalp have healed and closed; surgical treatment recommended; progressive worsening of symptoms, contraindicated for biological treatment
Yan et al., 2022 [6]	24-year-old male		KRT17 mutation	Unknown	Minocycline, Acitretin, Sulfasalazine, Celecoxib, 5-aminolevulinic acid-mediated interstitial photodynamic therapy (ALA-iPDT) with surgical incision and laser irradiation	Resolution of symptoms and almost-complete resolution of morphology with scarring noted after five months
Liu et al., 2024 [8]	50-year-old male		T2DM, Obesity, Genetic FOT History	Unknown	Ampicillin, Metronidazole, Debridement, Negative Pressure Drainage	Patient discharged and educated on wound care and healthy living habits
Lanocha et al., 2024 [9]	19-year-old male		Obesity, Hyperuricemia	Clindamycin, Gentamicin, Ichthammol	Doxycycline, Mupirocin, Rifampicin, Probiotic, Acetylsalicylic acid, Ichthammol, Intralesional methylprednisone	Positive response at 3 months; Methylprednisone led to resolution of skin lesions; treatment discontinued at 6 months and maintained on Clindamycin and Isotretinoin
Musumeci et al., 2019 [10]	24-year-old male		Overweight, KRT17 mutation	Unknown	Unknown	Unknown
Rahman et al., 2017 [11]	43-year-old male		T2DM, Obesity, Smoking	Surgical Procedure (Unspecified)	Unknown	Unknown
Vasanth et al., 2014 [12]	36-year-old male		Obesity	Unknown	Cephalosporin, Mupirocin, Isotretinoin	Lost to follow-up
Dhillon et al., 2012 [13]	42-year-old male		Smoking, Overweight	Multiple courses of antibiotics (unspecified); incision and drainage of lesions around anus	Clindamycin, Rifampicin, Finasteride	Partial response: clinical improvement between 3-12 months, relapse after 12 months.
Au et al., 2008 [14]	31-year-old male		Obesity	Multiple courses of antibiotics	Unknown	Unknown
Present Case	43-year-old female		Obesity, Hypertension	Ciprofloxacin, Spironolactone, Salicylic Acid	Attempt to start biologic treatment (secukinumab)	Unknown; lost to follow-up

Interpretation

Our case study is the first to report all four manifestations of the follicular occlusion tetrad in a female patient, as well as the first to observe symptom persistence after a glucagon-like peptide-1 (GLP-1) agonist. GLP-1 agonists bind to receptors in the brain, acting on the satiety centers [15]. They also attach to receptors in tissues, such as the pancreas and liver [15]. This binding allows for decreased glucagon/increased insulin uptake and decreased gluconeogenesis and inflammation, respectively [15]. These effects are important as obesity is a known shared factor that is linked to the development of HS and PC, two conditions found in the tetrad [11]. Obesity is linked to an increase in inflammatory cytokines, such as interleukin-6 (IL-6), IL-18, and tumor necrosis factor alpha (TNF-α), which are believed to play a role in HS and AC [2,7]. TNF-α inhibitors, such as adalimumab, have been used as treatments for HS and DC [2,7].

Nine cases have been previously reported of the full tetrad in the literature to date, although more cases have been reported of the follicular occlusion triad [5]. Treatment remains a challenge, with a risk of recurrence in numerous treatment modalities [5]. Our patient, like five others, sought care after previous treatment, explaining the need for newer therapies that may be more effective in managing the condition such as monoclonal antibodies and biologics.

Given the substantial association of obesity with the tetrad, it was interesting to observe symptom persistence in a patient actively using semaglutide for weight loss. It is important to note, however, that evidence for the efficacy of GLP-1 agonists and biologics in follicular occlusion tetrad is currently limited and mostly extrapolated from HS studies. Weight loss has also been previously noted to decrease the incidence of acne and HS [16]. However, hormonal imbalances induced by rapid weight loss, such as those associated with GLP-1 agonists, could potentially exacerbate symptoms of acne and acne-associated disorders by increasing sebum production as well [17]. GLP-1 agonists have been studied in HS patients, with further study needed in larger populations [15]. One study examined the role of liraglutide, with researchers finding decreased plasma cortisol and C-reactive protein (CRP) in addition to decreased weight circumference, suggesting less of a role for hormonal imbalance associated with weight loss. Decreased Hurley stage and Dermatology Quality Index (DLQI) were also noted, suggesting a potential positive role for patients with HS [18]. However, our patient did not see symptom relief, suggesting less efficacy in more complex presentations. The role of GLP-1 agonists has also been studied in acne patients, given the recent uptrend in patients reporting acne development shortly after GLP-1 agonist commencement [19]. However, no causal relationship through studies between acne development and GLP-1 agonists has been noted in the literature [19]. Further study is also warranted to determine the effect of management with secukinumab and bimekizumab, given its recent indications for treatment of HS [15,20].

## Conclusions

Follicular occlusion tetrad refers to diseases that share similar etiologies, including keratin rupture and chronic inflammation. Environmental and genetic factors, such as KRT17 mutations, have been associated with the development of the tetrad. This case highlights a rare example of a patient presenting with all four conditions while also managing weight loss through the use of semaglutide. Given the link between obesity and the development of the tetrad, it seems that the use of these treatments could potentially alleviate symptoms. It is important to clarify that evidence for the efficacy of GLP-1 agonists and biologics in follicular occlusion tetrad is currently limited and mostly extrapolated in HS studies. In this case, the patient’s symptoms persisted following weight loss treatment. Overall, the case presents significant opportunities for understanding the etiology and management of conditions encountered in practice through an uncommon manifestation.
